# Supplementary material for: Atrophic Gastritis and Autoimmunity: Results from a Prospective, Multicenter Study
Source: Diagnostics (Basel). 2023 Apr 30;13(9):1599. doi: 10.3390/diagnostics13091599 (PMC10178247; doi:10.3390/diagnostics13091599)
Supplement: Supplementary file 1 [file diagnostics-13-01599-s001.zip › diagnostics-2284261-supplementary.pdf]

Supplementary Materials

Table S1. Seropositivity of myositis antibodies in patients with CAG and control patients.

**Myositis-related antibody**	**AIG (*n* = 45)**	**NAIG (*n* = 109)**	**Control (*n* = 201)**	**Total (*n* = 355)**
Ro52 *n* (%)	1 (2.2)	1 (0.9)	3 (1.5)	5 (1.4)
OJ	0	2 (1.9)	0	2 (0.6)
EJ	0	0	0	0
PL.12	0	1 (0.9)	4 (2.0)	5 (1.4)
PL.7	1 (2.2)	1 (0.9)	2 (1.0)	4 (1.1)
SRP	1 (2.2)	2 (1.9)	0	3 (0.9)
Jo1	1 (2.2)	2 (1.9)	1 (0.5)	4 (1.1)
PM75	2 (4.4)	5 (4.7)	9 (4.5)	16 (4.5)
PM100	0	3 (2.8)	2 (1.0)	5 (1.4)
Ku	0	0	2 (1.0)	2 (0.6)
SAE1	0	1 (0.9)	0 (0.0)	1 (0.3)
NXP2	1 (2.2)	0	0	1 (0.3)
MDA5	0	0	1 (0.5)	1 (0.3)
TIF1g	1 (2.3)	0	1 (0.5)	2 (0.6)
Mi2a	0	0	0	0
Mi2b	0	1 (0.95)	1 (0.5)	2 (0.6)
At least one antibody equivocal or positive n (%)	7 (14.3)	15 (15.6)	26 (12.9)	48 (13.6)
At least one positive antibody n (%)	4 (8.9)	6 (5.5)	9 (4.4)	19 (5.3)

AIG, Autoimmune Gastritis; NAIG, Non-autoimmune Gastritis; Seropositivity for myositis-associated antibodies: negative <10, equivocal > 10, positive >25. For every antibody presented seropositivity is for >10, values are presented as n (%).

Table S2 ANA concentrations in patients with CAG and control patients.

**ANA**	**AIG (*n* = 45)**	**NAIG (*n* = 109)**	**p-value**	**Control (*n* = 201)**	**Total (*n* = 355)**
Negative	21 (46.7)	64 (59.8)		125 (62.5)	210 (59.7)
Equivocal	3 (6.7)	12 (11.2)		21 (10.5)	36 (10.2)
Weakly positive	10 (22.2)	14 (13.1)		21(10.5)	45 (12.8)
positive	10 (22.2)	13 (12.1)		26 (12.8)	49 (13.9)
Strongly positive	1 (2.2)	4 (3.7)		7 (3.5)	12 (3.4)
All positive results combined	**21 (46.7)**	**31 (29.0)**	**0.033**	54 (27.0)	106 (30.1)

AIG, Autoimmune Gastritis; NAIG, Non-autoimmune Gastritis; ANA, anti-nuclear antibodies; ANA results: negative dilution <1:80; equivocal 1:80; weakly positive 1:160; positive for 1:320 or 1:640; strongly positive for the dilution ≥1:1280. Values are presented as n (%).
